# Nutritional Markers and Perinatal Maternal Mental Health: A Network Analysis

**DOI:** 10.1192/j.eurpsy.2023.652

**Published:** 2023-07-19

**Authors:** J. van der Waerden, B. Knox, C. Galéra, A.-L. Sutter-Dallay, B. Heude, B. de Lauzon-Guillain

**Affiliations:** ^1^INSERM U1136-IPLESP/ERES; ^2^Sorbonne Université, Paris; ^3^ University of Bordeaux; ^4^INSERM, Bordeaux Population Health Center, UMR1219; ^5^Centre Hospitalier Perrens, Bordeaux; ^6^Université de Paris, CRESS, INSERM, INRAE, Paris, France

## Abstract

**Introduction:**

Perinatal maternal depression and anxiety are associated with adverse maternal outcomes, and nutrition may play an important role in their emergence. Previous research shows that certain micro and macronutrients found in different dietary patterns may influence perinatal mood disorders.

**Objectives:**

This study aims to explore relationships between nutrition during pregnancy and perinatal maternal depression and anxiety symptoms using network analyses.

**Methods:**

Using data from the French EDEN mother-child cohort, the sample consisted of 1438 women with available perinatal mental health outcomes (CES-D, STAI and EPDS) and nutritional markers collected from food frequency questionnaires. Four networks were constructed to explore the relationships between prenatal nutrient status, dietary patterns, and perinatal mental health, while accounting for important confounders.

**Results:**

The Healthy dietary pattern was associated with the presence of vital micronutrients, while the Western dietary pattern was consistently associated with poorer intake of vital micronutrients and contained an excess of certain macronutrients. Western dietary pattern and symptoms of postnatal depression were connected by a positive edge in both the macronutrient and micronutrient networks. Lower education levels were associated with higher Western dietary pattern scores, from which a positive edge linked to postnatal depression symptoms in both models.

**Conclusions:**

A Western dietary pattern was associated with increased symptoms of postnatal depression in our adjusted network models; The Healthy dietary pattern was associated with essential micronutrients but not with symptoms of depression or anxiety. Perinatal mental health might be impacted by specific dietary patterns in the context of psychosocial and physical stress associated with pregnancy.

**Disclosure of Interest:**

None Declared

